# Gender Inequality and Mental Health During the COVID-19 Pandemic

**DOI:** 10.3389/ijph.2021.1604220

**Published:** 2021-12-09

**Authors:** Florencia Borrescio-Higa, Patricio Valenzuela

**Affiliations:** ^1^ Business School, Universidad Adolfo Ibañez, Santiago, Chile; ^2^ Faculty of Engineering and Applied Sciences, Universidad de los Andes, Santiago, Chile

**Keywords:** mental health, gender, inequality, well-being, COVID-19

## Abstract

**Objective:** We explore gender differences in mental health deterioration and psychological well-being due to the COVID-19 pandemic, as well as the mechanisms through which these differences may operate.

**Methods:** Using data from the *Life during Pandemic* survey in Chile, which covers 2,545 adult respondents, we estimate econometric models to explore gender differences in psychological well-being and mental health as well as economic fragility and household workload during the COVID-19 pandemic.

**Results:** We find women are more likely to report overall bad mental health and deterioration of well-being. They are also more likely to have a new diagnosis of a mental health problem, to be pursuing treatment and taking prescription medication. Moreover, women report an increase in household chores and in childcare, and are more likely to have lost their employment or experienced a loss of income due to the pandemic.

**Conclusion:** Our results offer a general picture of gender differences in the psychological impact of COVID-19. We argue that policies that mitigate economic stress and address the needs of women specifically may ease mental health deterioration due to the pandemic.

## Introduction

The COVID-19 pandemic has produced an unprecedented and long-lasting shock to households around the world. Many countries have seen declines in their economic activity, leading to income loss and higher rates of unemployment and poverty. Some segments of the population are more vulnerable than others to the multidimensional effects of the pandemic. In particular, the pandemic has affected gender inequality by having a bigger impact on sectors with high female employment shares. Dang et al. [1] show that during the pandemic, women are more likely than men to permanently lose their jobs, and they expect their own labor income to decrease more in the future than men do. The pandemic has also affected gender inequality by increasing childcare needs in response to the closure of schools and daycare centers, which affects more mothers than fathers [2–4].

Correlated with economic concerns and food insecurity, mental health deteriorated significantly during the early months of the COVID-19 pandemic [5–7]. Specifically, high overall levels of depression, anxiety, and distress in countries such as United States, Canada, and United Kingdom have been reported [5, 8–12]. In light of the fact that women are more likely to present depressive symptoms in general [11], as well as to assume more housework and childcare activities than men [13, 14], a link between gender and mental health and well-being deterioration in the context of the pandemic is plausible.

Chile provides an interesting “laboratory” for the study of gender inequality during the COVID-19 pandemic. First, quarantine has had a large impact on economic activity in the country. Lockdowns were implemented locally and announced weekly [15]. Overall economic activity in Chile dropped significantly in 2020, and lockdowns have been strongly associated with a decrease in local economic activity [16]. Second, Chile’s female labor participation is one of the lowest among OECD countries, and the contribution of women in the workforce has decreased considerably during the pandemic. Female labor force participation went from 53.3 percent prior to the pandemic to 41.2 percent by June 2020 as sectors with high female employment participation, such as the service sector, were heavily affected [17].

This study contributes to the new body of research that explores how economic fragility and domestic chores are among the main stressors contributing to widespread emotional distress during the pandemic, and how some specific groups are more vulnerable than others to the emotional effects of pandemics [19]. Our results suggest the effects of the COVID-19 pandemic has worrying implications for women’s mental health and social functioning. Thus, policymakers and health care providers need to monitor the psychosocial needs of economically vulnerable women during periods of pandemic.

The aim of this study is to present a general picture of gender differences in the psychological impact of COVID-19 in Chile. The objective is to explore gender differences in mental health deterioration and psychological well-being due to the COVID-19 pandemic, as well as the mechanisms through which these differences may operate. Specifically, we explore the role of unemployment, income loss, increases in household chores, and increases in time spent caring for child or elders in the household in shaping the relationship between gender and mental health.

## Methods

### Study Design and Data

We use cross-sectional data from a nationwide large-scale survey *Vida en Pandemia* (Life during pandemic) survey, which has observations from all regions in Chile [20]. The survey was implemented July 13–17, 2020, through phone calls (i.e., 4.5 months after the first case of COVID-19 was detected in the country). The sample size is 2,545 adult respondents, 1,271 of whom identify as female, accounting for 50 percent of the sample. The survey was designed to be balanced in terms of age, gender, and municipality of residence, in order to render representativeness across these dimensions. Since the data are deidentified, this study of was not considered human subjects research.

The survey contains information on basic demographic variables of respondents, including age, gender, educational level, socioeconomic characteristics, and municipality of residence. Further, it contains detailed information on employment, economic hardship and financial health, living arrangements, and a series of measures on self-reported well-being.

The main outcome variables are indicators for poor well-being, sleeping problems, feeling distress, and feeling sadness. In all cases, we construct indicators that are equal to 1 if the individual reports frequently or very frequently experiencing these issues/feelings, and 0 otherwise. We also focus our analysis on a well-being deterioration variable that is equal to 1 if the individual reports that her well-being or mental health has worsened when compared to February (before the pandemic), and 0 otherwise. While the survey design is cross-sectional in nature, this variable allows to capture the change in well-being as reported by individuals. Another group of outcome variables are related to mental health care utilization, with indicators for a new diagnosis, new treatment, and new medication for a mental health condition after the start of the pandemic. There are also two indicators for economic fragility, unemployment and income loss. The former is equal to 1 if the individual reports unemployment after March 2020 as a direct consequence of the pandemic, and 0 otherwise. We create an indicator variable of income loss that is equal to 1 if the income reported in May is lower than the income reported for February, and 0 otherwise. Further, there are indicators for an increase in: 1) household chores, 2) childcare, and 3) care of elderly family members, with 1 representing an increase in comparison to the pre-pandemic early weeks of March, and 0 otherwise.

### Methodology

To examine the gender difference in the effect of the COVID-19 pandemic, we rely on the following linear probability model (LPM):

$$y_{ir}=A_{r}+\beta_{1}female_{i}+\beta_{2}X_{i}+\varepsilon_{i}$$

where $y_{ir}$ is a dependent variable of interest of individual i in region r , and $female_{i}$ is a dummy variable that equals 1 for women, and 0 otherwise. We include a set of control variables in the vector $X_{i}$ . Control variables include the following: age-category dummies (18–24, 25–34, 35–44, 45–54, 55–64, and over 65); an indicator for household head; education-achievement indicators (complete or incomplete primary, secondary, technical, bachelor’s, and master’s level or more); an indicator for living with a partner (married or cohabiting); indicators for the presence of young children (under the age of 12) and for the presence of elders (over the age of 65) in the household; and indicators for private health insurance, public-sector employment, self-employment, and migrant status. In most specifications we also control for pre-pandemic household income (expressed in natural logarithm). All regressions include a set of region indicators ( $A_{r}$ ) to account for fixed characteristics at the region level. Standard errors are robust to heteroskedasticity.

We are interested in the coefficient on the  female  variable, which measures the gender gap between men and women in our different outcome variables related to well-being, mental health, economic fragility, and household workload.

## Results

Using our sample of 2,545 adults in Chile, in this section we report the prevalence of self-reported well-being, utilization of mental health care services, economic fragility, and increase in household workload. Columns 1 and 2 in Table 1 present the median and standard deviation of each (dichotomous) outcome variable for the full sample. We report descriptive statistics characterizing survey respondents by gender in Table 2. Columns 1 and 2 report the median and standard deviation of each variable for women (1,271 observations), and columns 3 and 4 report the median and standard deviation of each variable for men (1,274 observations). Column 5 reports the difference between the means, and column 6 reports the *p*-value of the mean difference. Some significant differences exist between women and men. Specifically, men in the sample are slightly older and are more likely to report being the household head. Women are less likely to report living with a partner but are more likely to have young children in the household. As expected in this context, women are less likely to be employed before the pandemic.

**TABLE 1:** Descriptive statistics outcome variables.

	Mean	Std. Dev.	Min	Max
Well-being				
Poor wellbeing	0.337	0.473	0	1
Sleeping problems	0.431	0.495	0	1
Deterioration	0.555	0.497	0	1
Distress	0.387	0.487	0	1
Sadness	0.345	0.476	0	1
Mental health				
Diagnosis	0.044	0.204	0	1
Treatment	0.036	0.187	0	1
Medication	0.043	0.202	0	1
Economic fragility				
Unemployment	0.333	0.471	0	1
Income loss	0.378	0.485	0	1
Household workload				
Household chores	0.684	0.465	0	1
Childcare	0.199	0.399	0	1
Elderly care	0.070	0.255	0	1

This table reports simple descriptive statistics (mean, standard deviation, minimum value and maximum value) for all the outcome variables. The outcome variables are divided into four groups: well-being, mental health, economic fragility and increase in household workload.Vida en Pandemia, Chile 2020.

**TABLE 2:** Descriptive statistics control variables by gender.

	(1)		(2)		(3)	
	Female		Male		Difference	
	Mean	Std. Dev.	Mean	Std. Dev.	B	p
Age	43.777	15.149	45.744	16.554	1.968**	(0.002)
Household head	0.411	0.492	0.648	0.478	0.237***	(0.000)
Primary–incomplete	0.002	0.049	0.002	0.048	−0.000	(0.998)
Primary–complete	0.005	0.069	0.005	0.074	0.001	(0.784)
Secondary–incomplete	0.042	0.200	0.031	0.174	−0.010	(0.166)
Secondary–complete	0.154	0.361	0.148	0.355	−0.007	(0.640)
Technical–incomplete	0.054	0.225	0.055	0.228	0.001	(0.872)
Technical–complete	0.187	0.390	0.153	0.360	−0.034*	(0.022)
Bachelor–incomplete	0.136	0.343	0.142	0.349	0.006	(0.664)
Bachelor–complete	0.341	0.474	0.356	0.479	0.016	(0.407)
Master or more	0.079	0.271	0.107	0.309	0.027*	(0.018)
Living with a partner	0.482	0.500	0.586	0.493	0.103***	(0.000)
Young children in household	0.326	0.469	0.246	0.431	−0.079***	(0.000)
Elder in household	0.196	0.397	0.202	0.401	0.006	(0.713)
Private health insurance	0.290	0.454	0.349	0.477	0.059**	(0.001)
Public sector	0.158	0.365	0.124	0.330	−0.034*	(0.013)
Self-employed	0.205	0.404	0.214	0.410	0.009	(0.579)
Migrant	0.049	0.215	0.076	0.265	0.027**	(0.004)
Employed pre-pandemic	0.778	0.416	0.831	0.375	0.053***	(0.001)
Pre-pandemic income	1.101	1.112	1.159	1.024	0.058	(0.231)
Observations	1271		1274		2545	

This table reports simple descriptive statistics (mean and standard deviation) for all the control variables by gender. The table also reports the mean difference (and the p-value) between men and women. Pre-pandemic income is measured in 1000s Chilean pesos.Vida en Pandemia, Chile 2020.

### Multivariate Analysis

#### Gender Differences in Well-Being and Mental Health

We explore whether significant gender differences exist in terms of well-being and mental health during the pandemic period, after controlling for a comprehensive set of variables. The results in Table 3 suggest that during the pandemic, women are more likely to report poor well-being (columns 1 and 2), have had more sleeping problems (columns 3 and 4), and experience a stronger deterioration in their mood in comparison to the pre-pandemic period (columns 5 and 6). They are also more likely to experience negative emotions such as distress (columns 7 and 8) and sadness (columns 9 and 10). Note that some specifications control for the pre-pandemic labor income, and thus are restricting the sample to those who had labor income in February (pre-pandemic). Essentially, we compare working women and men who were working in February, we find that women are more likely to have worse mental health and well-being outcomes, after controlling for income and other characteristics. Supplementary Table A1 in the Supplementary Material estimates our specification including all the controls for the full set of negative emotions asked in the survey: restlessness, fear, sadness, overwhelmed, hopelessness, frustration, fear, worry, pessimism, and anger. The results are consistent with our main findings.

**TABLE 3:** Well-being.

	(1)	(2)	(3)	(4)	(5)	(6)	(7)	(8)	(9)	(10)
	Poor well-being		Sleeping problems		Deterioration		Distress		Sadness	
Female	0.0646***	0.0546**	0.109***	0.104***	0.105***	0.0887***	0.122***	0.111***	0.117***	0.105***
	[0.0200]	[0.0229]	[0.0206]	[0.0236]	[0.0205]	[0.0236]	[0.0202]	[0.0234]	[0.0200]	[0.0230]
Age (18–24)	0.161***	0.214***	0.298***	0.290***	0.181***	0.231***	0.328***	0.337***	0.199***	0.166***
	[0.0448]	[0.0567]	[0.0459]	[0.0567]	[0.0468]	[0.0570]	[0.0448]	[0.0562]	[0.0453]	[0.0567]
Age (25–34)	0.153***	0.160***	0.162***	0.179***	0.162***	0.170***	0.225***	0.233***	0.108***	0.120***
	[0.0339]	[0.0393]	[0.0350]	[0.0402]	[0.0368]	[0.0426]	[0.0336]	[0.0390]	[0.0336]	[0.0382]
Age (35–44)	0.0973***	0.0944**	0.125***	0.138***	0.0814**	0.0892**	0.150***	0.138***	0.0650*	0.0807**
	[0.0361]	[0.0410]	[0.0374]	[0.0423]	[0.0393]	[0.0447]	[0.0360]	[0.0408]	[0.0359]	[0.0403]
Age (45–54)	0.0907***	0.100**	0.130***	0.134***	0.108***	0.132***	0.129***	0.135***	0.0447	0.0619
	[0.0340]	[0.0391]	[0.0353]	[0.0404]	[0.0371]	[0.0429]	[0.0338]	[0.0392]	[0.0333]	[0.0378]
Age (55–64)	−0.00278	−0.0199	0.0789**	0.0782**	0.0570	0.0487	0.0689**	0.0489	0.0281	0.0394
	[0.0323]	[0.0364]	[0.0344]	[0.0390]	[0.0368]	[0.0423]	[0.0325]	[0.0370]	[0.0326]	[0.0364]
Household head	0.0323	0.0334	−0.0133	−0.0285	−0.0220	−0.0208	0.0252	0.0107	−0.000415	−0.0218
	[0.0215]	[0.0246]	[0.0222]	[0.0255]	[0.0221]	[0.0253]	[0.0218]	[0.0251]	[0.0215]	[0.0248]
Primary–incomplete	−0.104	−0.131	0.00262	−0.0564	0.0578	−0.000421	0.192	0.116	0.224	0.187
	[0.164]	[0.166]	[0.232]	[0.234]	[0.206]	[0.212]	[0.180]	[0.178]	[0.206]	[0.210]
Primary–complete	0.0193	0.0721	−0.104	−0.0714	−0.198	−0.201	0.00273	−0.0559	0.0818	−0.0358
	[0.136]	[0.174]	[0.137]	[0.166]	[0.125]	[0.150]	[0.134]	[0.156]	[0.138]	[0.175]
Secondary–incomplete	0.0234	0.0205	0.107*	0.108	−0.0382	−0.0483	0.0525	0.0239	0.0722	0.115*
	[0.0584]	[0.0668]	[0.0592]	[0.0678]	[0.0611]	[0.0697]	[0.0594]	[0.0677]	[0.0594]	[0.0690]
Secondary–complete	0.0119	−0.00529	0.0842**	0.0143	0.0676	0.0182	0.0549	−0.00708	0.0434	0.0167
	[0.0404]	[0.0462]	[0.0412]	[0.0477]	[0.0427]	[0.0499]	[0.0401]	[0.0471]	[0.0396]	[0.0461]
Technical–incomplete	−0.0274	−0.0241	0.114**	0.0686	0.0352	0.00973	0.0560	0.0403	0.113**	0.114*
	[0.0518]	[0.0598]	[0.0537]	[0.0624]	[0.0552]	[0.0642]	[0.0522]	[0.0604]	[0.0529]	[0.0605]
Technical–complete	−0.0229	−0.0340	0.0589	0.0328	0.0206	−0.0287	0.0443	−0.00578	0.0176	−0.00766
	[0.0391]	[0.0452]	[0.0400]	[0.0460]	[0.0414]	[0.0480]	[0.0390]	[0.0452]	[0.0382]	[0.0440]
Bachelor–incomplete	0.0263	0.0360	0.110***	0.0829*	0.109**	0.0896*	0.0748*	0.0456	0.0843**	0.0733
	[0.0422]	[0.0482]	[0.0425]	[0.0489]	[0.0436]	[0.0499]	[0.0418]	[0.0480]	[0.0411]	[0.0470]
Bachelor–complete	0.000593	−0.00108	0.0318	0.00312	0.0788**	0.0498	0.0281	−0.00812	0.00873	−0.00425
	[0.0342]	[0.0373]	[0.0347]	[0.0387]	[0.0363]	[0.0405]	[0.0336]	[0.0375]	[0.0330]	[0.0364]
Living with a partner	−0.0372*	−0.0183	0.00143	0.00650	0.0189	0.0413*	−0.0213	−0.00640	−0.0545**	−0.0511**
	[0.0212]	[0.0245]	[0.0218]	[0.0251]	[0.0218]	[0.0251]	[0.0214]	[0.0247]	[0.0213]	[0.0246]
Children in household	0.0148	−0.00185	0.0676***	0.0613**	0.0754***	0.0599**	0.00225	−0.0119	0.0279	0.0346
	[0.0229]	[0.0262]	[0.0233]	[0.0267]	[0.0231]	[0.0265]	[0.0230]	[0.0264]	[0.0227]	[0.0262]
Elder in household	−0.0207	−0.0425	0.00722	−0.00213	0.00391	0.0133	−0.00294	0.00181	−0.00538	−0.00260
	[0.0237]	[0.0271]	[0.0245]	[0.0285]	[0.0253]	[0.0297]	[0.0244]	[0.0282]	[0.0242]	[0.0278]
Private health insurance	−0.00671	0.0139	0.00164	0.00573	0.0354	0.0473*	0.00905	0.0424	−0.0125	0.00363
	[0.0217]	[0.0263]	[0.0226]	[0.0272]	[0.0228]	[0.0278]	[0.0224]	[0.0272]	[0.0217]	[0.0260]
Public sector	0.0529*	0.0856***	−0.0348	−0.0260	0.00534	0.0335	0.0309	0.0568*	0.0229	0.0495
	[0.0288]	[0.0330]	[0.0294]	[0.0336]	[0.0292]	[0.0329]	[0.0292]	[0.0334]	[0.0285]	[0.0325]
Self-employed	0.0108	0.00637	−0.0100	−0.00835	0.00819	0.00668	0.00103	−0.0152	0.00623	0.00583
	[0.0233]	[0.0267]	[0.0239]	[0.0276]	[0.0245]	[0.0284]	[0.0235]	[0.0268]	[0.0233]	[0.0269]
Migrant	−0.0800**	−0.0914**	−0.0828**	−0.0989**	−0.112***	−0.130***	−0.0398	−0.0551	0.00439	−0.0160
	[0.0364]	[0.0414]	[0.0390]	[0.0439]	[0.0421]	[0.0478]	[0.0385]	[0.0436]	[0.0382]	[0.0424]
Pre-pandemic income (log)		−0.104***		−0.0679**		−0.0717**		−0.128***		−0.0894***
		[0.0317]		[0.0330]		[0.0339]		[0.0335]		[0.0318]
Observations	2,545	1,943	2,545	1,943	2,545	1,943	2,545	1,943	2,545	1,943
R-squared	0.039	0.052	0.065	0.068	0.060	0.061	0.066	0.078	0.056	0.062

This table reports estimates from a Linear Probability Model (LPM) of the probability of an individual reporting poor well-being, sleep problems, deterioration of well-being, or negative feelings against the variables listed below. All regressions control for region dummy variables. Heteroskedasticity-robust standard errors are in parentheses. ***, **, * indicate significance at the 1%, 5% and 10% levels, respectively.Vida en Pandemia, Chile 2020.

Table 3 presents other interesting findings. Younger adults show worse levels of well-being relative than over the age of 65. The presence of young children is positively associated with sleeping problems and the deterioration of well-being. Finally, pre-pandemic income is negatively associated with all our outcome variables.

#### Gender Differences in Diagnosis and Treatment of Mental Health Problems

We examine whether significant gender differences exist in terms of the utilization of mental health care services during the pandemic, by analyzing whether individuals report having a new diagnosis and/or a new treatment of a mental health problem. Columns 1 and 2 in Table 4 suggest women are more likely than men to have a new diagnosis of a mental health problem, and they are also more likely to report being in treatment. Column 3 indicates women are more likely to use mental health medication than men during the pandemic, although this result is only significant at the 10% level.

**TABLE 4:** Mental health diagnosis and treatment.

	(1)	(2)	(3)
	Diagnosis	Treatment	Medication
Female	0.0197**	0.0287***	0.0178*
	[0.00986]	[0.00920]	[0.00995]
Age (18–24)	0.0220	0.0396**	0.00260
	[0.0230]	[0.0194]	[0.0206]
Age (25–34)	0.0146	0.0296**	0.00342
	[0.0147]	[0.0116]	[0.0144]
Age (35–44)	0.0343*	0.0440***	0.0276
	[0.0179]	[0.0149]	[0.0181]
Age (45–54)	0.00636	0.0171	−0.00153
	[0.0160]	[0.0121]	[0.0151]
Age (55–64)	0.0186	0.0233**	0.0180
	[0.0158]	[0.0118]	[0.0163]
Household head	0.0144	0.00395	−0.00456
	[0.0110]	[0.00955]	[0.0109]
Primary–incomplete	−0.0267	−0.0223	−0.0488**
	[0.0213]	[0.0205]	[0.0241]
Primary–complete	0.108	0.101	0.0853
	[0.120]	[0.120]	[0.119]
Secondary–incomplete	0.0587*	0.0625*	0.00794
	[0.0348]	[0.0341]	[0.0320]
Secondary–complete	0.0404**	0.0127	−0.00706
	[0.0197]	[0.0183]	[0.0202]
Technical–incomplete	0.0297	−0.00252	0.00310
	[0.0255]	[0.0203]	[0.0272]
Technical–complete	0.0339*	0.0101	−0.0107
	[0.0186]	[0.0178]	[0.0196]
Bachelor–incomplete	0.0199	0.0178	0.00103
	[0.0187]	[0.0199]	[0.0222]
Bachelor–complete	0.000292	−0.00199	−0.0165
	[0.0140]	[0.0159]	[0.0185]
Living with a partner	0.0120	0.0161*	0.00878
	[0.0110]	[0.00960]	[0.0106]
Children in household	0.00399	−0.00829	−0.000840
	[0.0119]	[0.0108]	[0.0109]
Elder in household	0.00971	−0.00625	0.00347
	[0.0120]	[0.00932]	[0.0116]
Private health insurance	0.0215*	0.0106	0.0302**
	[0.0113]	[0.0109]	[0.0124]
Public sector	0.0298*	0.0363**	0.0247
	[0.0163]	[0.0162]	[0.0160]
Self-employed	−0.00391	0.00460	0.00739
	[0.0103]	[0.00953]	[0.0111]
Migrant	−0.00383	0.0331	−0.00634
	[0.0170]	[0.0209]	[0.0166]
Pre-pandemic income (log)	−0.00858	0.0241*	0.00821
	[0.0137]	[0.0141]	[0.0126]
Observations	1,943	1,943	1,943
R-squared	0.024	0.040	0.026

This table reports estimates from a Linear Probability Model (LPM) of the probability of an individual reporting a new mental health diagnosis, new treatment or new medication (during the pandemic) against the variables listed below. All regressions control for region dummy variables. Heteroskedasticity-robust standard errors are in parentheses. ***, **, * indicate significance at the 1%, 5% and 10% levels, respectively.Vida en Pandemia, Chile 2020.

#### Gender Differences in Economic Fragility and Household Workload

We present the potential mechanisms through which women present higher levels of deterioration of their psychological well-being during the pandemic than do men in Table 5. Columns 1 and 2 examine whether women are more likely to experience unemployment and income losses during the pandemic period. We restrict our sample to those individuals who were employed in the pre-pandemic period (i.e., February 2020). Column 1 shows women are more likely than men to become unemployed because of the COVID-19 pandemic. Column 2 shows women are also more likely to report a loss in income. These results confirm the pandemic’s toll on employment and income has been heavier for women, and therefore, women are more economically vulnerable to negative shocks.

**TABLE 5:** Economic fragility and household workload.

	(1)	(2)	(3)	(4)	(5)
	Unemployment	Income loss	Household chores	Childcare	Elderly care
Female	0.0476*	0.0695***	0.0417*	0.0393***	0.0163
	[0.0247]	[0.0251]	[0.0225]	[0.0121]	[0.0115]
Age (18–24)	0.238***	0.102	−0.0133	0.0695***	0.0390
	[0.0702]	[0.0730]	[0.0565]	[0.0259]	[0.0300]
Age (25–34)	0.184***	0.108**	0.0800**	0.0627***	0.0470**
	[0.0465]	[0.0522]	[0.0395]	[0.0172]	[0.0227]
Age (35–44)	0.132***	0.0719	0.0483	0.0568***	0.0474**
	[0.0482]	[0.0537]	[0.0416]	[0.0206]	[0.0235]
Age (45–54)	0.108**	0.0580	0.0320	0.0269	0.0497**
	[0.0467]	[0.0523]	[0.0403]	[0.0190]	[0.0229]
Age (55–64)	0.148***	0.0741	0.0419	0.0213	0.0675***
	[0.0472]	[0.0517]	[0.0389]	[0.0141]	[0.0238]
Household head	−0.0187	−0.0152	0.0202	0.0355***	0.0149
	[0.0267]	[0.0278]	[0.0239]	[0.0130]	[0.0121]
Primary–incomplete	−0.238	0.0760	−0.266	0.0532	0.0481
	[0.200]	[0.214]	[0.210]	[0.0523]	[0.128]
Primary–complete	0.193	0.0156	−0.129	−0.127	−0.0634*
	[0.195]	[0.158]	[0.174]	[0.124]	[0.0364]
Secondary–incomplete	0.214***	0.161**	−0.0804	0.0123	0.0507
	[0.0727]	[0.0751]	[0.0647]	[0.0316]	[0.0408]
Secondary–complete	0.0821*	0.138***	−0.125***	−0.00550	−0.0130
	[0.0495]	[0.0513]	[0.0454]	[0.0232]	[0.0216]
Technical–incomplete	0.00845	0.165**	−0.179***	−0.0294	0.0259
	[0.0631]	[0.0648]	[0.0605]	[0.0370]	[0.0298]
Technical–complete	0.0269	0.0991**	−0.131***	−0.0146	−0.0119
	[0.0454]	[0.0476]	[0.0430]	[0.0231]	[0.0197]
Bachelor–incomplete	0.0360	0.148***	−0.0607	0.00409	0.00786
	[0.0505]	[0.0532]	[0.0449]	[0.0218]	[0.0235]
Bachelor–complete	0.000619	0.0442	−0.0811**	−0.00947	−0.00745
	[0.0368]	[0.0401]	[0.0354]	[0.0178]	[0.0164]
Living with a partner	0.0320	−0.00631	0.0227	0.0140	−0.0126
	[0.0265]	[0.0269]	[0.0237]	[0.0127]	[0.0121]
Children in household	−0.0386	−0.0306	0.00127	0.692***	−0.00706
	[0.0276]	[0.0288]	[0.0258]	[0.0202]	[0.0123]
Elder in household	0.0401	0.0213	−0.000452	−0.00406	0.284***
	[0.0318]	[0.0331]	[0.0275]	[0.0129]	[0.0246]
Private health insurance	−0.0912***	−0.139***	0.0647**	0.00238	−0.0181
	[0.0277]	[0.0290]	[0.0263]	[0.0133]	[0.0128]
Public sector	−0.0930***	−0.131***	0.0215	0.0225	0.00403
	[0.0295]	[0.0316]	[0.0315]	[0.0177]	[0.0137]
Self-employed	0.125***	0.155***	0.0440*	−0.00543	0.00196
	[0.0295]	[0.0302]	[0.0264]	[0.0142]	[0.0146]
Migrant	0.0353	−0.0255	−0.0354	−0.00348	0.0222
	[0.0483]	[0.0462]	[0.0451]	[0.0238]	[0.0238]
Pre-pandemic income (log)	−0.122***	0.239***	−0.0407	0.000918	−0.0166
	[0.0348]	[0.0363]	[0.0313]	[0.0152]	[0.0172]
Observations	1,626	1,626	1,943	1,943	1,943
R-squared	0.102	0.095	0.032	0.642	0.202

This table reports estimates from a Linear Probability Model (LPM) of the probability of an individual reporting unemployment, income loss, an increase in household chores, an increase in childcare, or an increase in care of elders against the variables listed below. All regressions control for region dummy variables. Heteroskedasticity-robust standard errors are in parentheses. ***, **, * indicate significance at the 1%, 5% and 10% levels, respectively. Vida en Pandemia, Chile 2020.

Columns 3 to 5 explore whether women report a stronger increase in household chores, childcare, and care of elders than men during the pandemic period. These results show women are more likely to report an increase in household chores and an increase in childcare activities, though we find no significant differential effect in time allocated to caring for an elder in the household.

Overall, the results suggest women are more likely to report overall bad mental health across a variety of outcomes, even after we control for a comprehensive set of variables. We identify two channels that may be driving our main results. We find women are more likely to suffer unemployment and income loss because of the COVID-19 pandemic. Moreover, we find women are more likely to exhibit an increase in housework and childcare responsibilities.

#### Economic Fragility, Household Workload and Well-Being

So far we have explored gender differences in well-being and mental health, economic fragility and household workload. In this section, we move further and explore the nexus between economic fragility and well-being and between household workload and well-being. Our results are reported in Table 6. Specifically, columns 1 to 5 explore the relationship between well-being deterioration and unemployment, income loss, household chores, childcare and elderly care, respectively.

**TABLE 6:** Well-being deterioration, economic fragility and household workload.

Deterioration	(1)	(2)	(3)	(4)	(5)
Female	0.0830***	0.0830***	0.0848***	0.0864***	0.0867***
	[0.0233]	[0.0235]	[0.0236]	[0.0237]	[0.0236]
Age (18–24)	0.186***	0.215***	0.232***	0.226***	0.226***
	[0.0566]	[0.0572]	[0.0568]	[0.0572]	[0.0570]
Age (25–34)	0.135***	0.147***	0.163***	0.167***	0.165***
	[0.0430]	[0.0430]	[0.0426]	[0.0428]	[0.0426]
Age (35–44)	0.0603	0.0702	0.0846*	0.0859*	0.0834*
	[0.0447]	[0.0448]	[0.0444]	[0.0448]	[0.0446]
Age (45–54)	0.106**	0.116***	0.129***	0.130***	0.126***
	[0.0429]	[0.0430]	[0.0427]	[0.0429]	[0.0428]
Age (55–64)	0.0190	0.0336	0.0447	0.0474	0.0404
	[0.0423]	[0.0424]	[0.0420]	[0.0424]	[0.0423]
Household head	−0.0204	−0.0195	−0.0227	−0.0229	−0.0226
	[0.0251]	[0.0252]	[0.0252]	[0.0253]	[0.0253]
Primary–incomplete	0.0428	−0.00811	0.0247	−0.00357	−0.00629
	[0.199]	[0.207]	[0.204]	[0.210]	[0.204]
Primary–complete	−0.219	−0.203	−0.189	−0.193	−0.193
	[0.134]	[0.155]	[0.145]	[0.153]	[0.149]
Secondary–incomplete	−0.0740	−0.0744	−0.0407	−0.0490	−0.0545
	[0.0692]	[0.0696]	[0.0699]	[0.0695]	[0.0692]
Secondary–complete	0.0132	0.00193	0.0300	0.0185	0.0198
	[0.0493]	[0.0498]	[0.0498]	[0.0499]	[0.0499]
Technical–incomplete	0.0179	−0.00854	0.0266	0.0115	0.00657
	[0.0635]	[0.0645]	[0.0639]	[0.0638]	[0.0639]
Technical–complete	−0.0286	−0.0415	−0.0163	−0.0278	−0.0273
	[0.0473]	[0.0479]	[0.0479]	[0.0480]	[0.0480]
Bachelor–incomplete	0.0864*	0.0741	0.0953*	0.0894*	0.0886*
	[0.0494]	[0.0500]	[0.0498]	[0.0499]	[0.0501]
Bachelor–complete	0.0539	0.0448	0.0575	0.0504	0.0507
	[0.0400]	[0.0405]	[0.0403]	[0.0405]	[0.0405]
Living with a partner	0.0387	0.0426*	0.0392	0.0405	0.0429*
	[0.0248]	[0.0249]	[0.0249]	[0.0251]	[0.0250]
Children in household	0.0636**	0.0642**	0.0598**	0.0190	0.0608**
	[0.0262]	[0.0264]	[0.0265]	[0.0410]	[0.0266]
Elder in household	0.00921	0.0106	0.0133	0.0135	−0.0214
	[0.0294]	[0.0296]	[0.0294]	[0.0297]	[0.0327]
Private health insurance	0.0594**	0.0597**	0.0412	0.0471*	0.0495*
	[0.0276]	[0.0276]	[0.0277]	[0.0278]	[0.0278]
Public sector	0.0456	0.0452	0.0314	0.0321	0.0330
	[0.0330]	[0.0330]	[0.0328]	[0.0329]	[0.0328]
Self-employed	−0.0180	−0.0184	0.00252	0.00700	0.00644
	[0.0283]	[0.0285]	[0.0284]	[0.0284]	[0.0284]
Migrant	−0.135***	−0.128***	−0.126***	−0.129***	−0.132***
	[0.0478]	[0.0477]	[0.0477]	[0.0477]	[0.0474]
Pre-pandemic income (log)	−0.0495	−0.0997***	−0.0678**	−0.0717**	−0.0697**
	[0.0338]	[0.0342]	[0.0337]	[0.0339]	[0.0337]
Unemployment	0.160***				
	[0.0240]				
Income loss		0.120***			
		[0.0234]			
Household chores			0.0946***		
			[0.0243]		
Childcare				0.0592	
				[0.0442]	
Elderly care					0.122**
					[0.0484]
Observations	1,943	1,943	1,943	1,943	1,943
R-squared	0.082	0.074	0.069	0.062	0.064

This table reports estimates from a Linear Probability Model (LPM) of the probability of an individual reporting deterioration of well-being against the variables listed below. All regressions control for region fixed effects. Heteroskedasticity-robust standard errors are in parentheses. ***, **, * indicate significance at the 1%, 5% and 10% levels, respectively. Vida en Pandemia, Chile 2020.

The results reported in Table 6 indicate that mental health deterioration (our preferred outcome variable) is positively related to economic fragility (as measured by unemployment and income loss) and household workload (household chores and care of elders in the household). In all these cases the coefficients are statistically significant, and the magnitudes are economically meaningful. We replicate this analysis in Supplementary Table A2 in the Supplementary Material by using poor-well-being and sleeping problems as dependent variables. Most of our previous findings remain qualitatively unchanged.

## Discussion

The COVID-19 pandemic is not only a global health emergency that is leading to a major economic downturn, but also has a tremendous psychological cost [21]. Recent reviews of the literature note a high prevalence of symptoms of depression, anxiety, post-traumatic stress disorder, and stress in several countries, and many point to the fact that women are more likely than men to exhibit these symptoms [5, 10, 11, 22].

Recent evidence presents two main explanations for the effect of the pandemic on gender inequality. One emphasizes that the pandemic has had a large impact on sectors with high female employment shares. Unlike other modern crises, the pandemic recession has led to more job and income losses among women than among men [2, 18, 23, 24]. Whereas most recent crises have hit industries dominated by men (e.g., construction), the recession caused by the coronavirus is having a greater effect on industries dominated by women (e.g., hospitality and retail). A second explanation, based on evidence from developed countries, emphasizes that the pandemic has increased not only housework, but also family responsibilities, including childcare needs primarily conducted by women in response to school closures [2, 4, 13, 14, 25, 26]. However, evidence of heterogeneity is apparent in the impact of the pandemic on gender inequality across countries, due to differences in female participation in the labor force [1].

This study sought to survey mental health deterioration and psychological well-being in Chile during the COVID-19 pandemic, with the aim of analyzing gender differences and identifying potential mechanisms. Overall, our results show women are more likely to report worse levels of mental health and well-being and higher levels of mental health deterioration than are men, even after controlling for pre-pandemic income, education, age, and the presence of young children in the household. These results are consistent with findings for other countries [4–6, 8, 14, 26]. These results show a cumulative vulnerability to the pandemic, since there is evidence of previous gender disparities in mental health. While the prevalence of depression in 2017 was 10.1 percent among women, it was 2.1 percent among men [27]. Other data sources point in the same direction, as the prevalence of depressive symptoms is almost double for adult women, 22.5 percent, compared to 12.9 percent for men [28].

Further, women are also more likely to have a new (i.e., during the pandemic) diagnosis for a mental health problem, and to be pursuing new treatment and taking medication. The low levels of mental health care service utilization during the pandemic are particularly worrying. Only 5 percent of women and less than 3 percent of men report having a new diagnosis or treatment of a mental health condition. Given the results on well-being, these percentages point to a lack of access, which is presumably related to the fact that during the lockdown, access to psychological health services was relatively difficult, and adjustment to the widespread use of telemedicine took time.

The deterioration in mental health and well-being, as well as an increase in sleeping problems, seems related to the fact that the pandemic’s toll on employment and income has been heavier for women, as has the pressure of housework, childcare, and homeschooling for those households with young children [3].

### Limitations

This analysis has limitations. The cross-sectional nature of the data limits the range of conclusions that can be drawn, especially in terms of dynamics. However, it does reflect the mental health and psychological well-being of respondents in the midst of a widespread lockdown when few mitigating policies were in effect, and some of the main outcome variables are expressed as changes with respect to before the pandemic. Another limitation is that this survey over-represents working women. However, the literature has shown that the Covid-19 pandemic has been particularly hard on working women and is therefore a relevant dimension of analysis. Further, the mental health and well-being variables used in the survey were not designed as clinical instruments. Future research should focus on the trajectory of mental health and well-being and on the effect of policies aimed at attenuating the effect of the economic crisis.

### Conclusion

Our results offer a general picture of gender differences in the psychological impact of COVID-19 in Chile. We find higher rates of the deterioration of mental health and psychological well-being for women than for men, and these rates are related to unemployment, loss of income, and an increase in housework and childcare. These results point to intersecting vulnerabilities, as gender roles and economic fragility interact creating unique challenges for women during the pandemic. We argue that policies that mitigate economic stress and address the needs of women specifically may ease the worsening of mental health due to the pandemic. Further, access to mental health services needs improvement, with the aim of preventing further mental illness.
