# Supplementary material for: Gender Inequality and Mental Health During the COVID-19 Pandemic
Source: Int J Public Health. 2021 Dec 9;66:1604220. doi: 10.3389/ijph.2021.1604220 (PMC8698135; doi:10.3389/ijph.2021.1604220)
Supplement: Supplementary file 1 [file DataSheet1.docx]

**SUPPLEMENTARY MATERIAL**

**Table A1**

**Additional Measures of Well-being**

This table reports estimates from a Linear Probability Model (LPM) of the probability of an individual reporting deterioration of well-being against the variables listed below. All regressions control for region fixed effects. Heteroskedasticity-robust standard errors are in parentheses. ***, **, * indicate significance at the 1%, 5% and 10% levels, respectively

*Gender Inequality and Mental Health during the COVID-19 Pandemic, Chile 2020*

**Table A2**

**Well-Being, Economic Fragility and Household Workload**

This table reports estimates from a Linear Probability Model (LPM) of the probability of an individual reporting poor well-being or sleeping problems against the variables listed below. All regressions control for region fixed effects. Heteroskedasticity-robust standard errors are in parentheses. ***, **, * indicate significance at the 1%, 5% and 10% levels, respectively. *Gender Inequality and Mental Health during the COVID-19 Pandemic, Chile 2020*
